# Eliciting the views of autistic adolescents attending specialist schools on what friendships mean to them

**DOI:** 10.1177/13623613251414302

**Published:** 2026-01-31

**Authors:** Jo Halsall, Elise Robinson, Anna Cook, Adam Halsall, Laura Crane

**Affiliations:** 1 University College London, UK; 2 West Sussex Educational Psychology Service, UK; 3 The Queensmill Trust, UK; 4 University of Surrey, UK; 5 Independent Researcher, UK; 6 University of Birmingham, UK

**Keywords:** autism, friendship, special educational needs, specialist schools

## Abstract

Friendship has been identified as an important way to support autistic adolescents’ social, emotional and cognitive development. However, research examining autistic adolescents’ friendships has tended to use methodologies inaccessible to children with complex communication and learning needs. We examined the friendship experiences of 12 autistic adolescents (aged 12–15 years) with complex communication and learning needs who attended a specialist school. Using reflexive thematic analysis, we identified two themes. Within the first theme of ‘reconceptualising friendship’, the adolescents identified friends who evoked strong emotions and connection, without distinguishing between positive or negative interactions, or the standard boundaries of friendship. ‘Friendships’ with adults were also highlighted. Within the second theme of ‘the context dependent nature of connection’, the adolescents’ descriptions of friendship communicated a preference for engagement via physical play in spaces that facilitated such interaction. Our findings underscore the importance of directly eliciting the views of autistic adolescents with complex communication and learning needs. They also highlight the value of facilitating opportunities to build social connection with peers and adults through physical play and activities that provoke strong emotions within a safe and contained context.

**Lay abstract:**

Friendship plays a key role in helping autistic young people develop: socially, emotionally and cognitively. However, much of the research on friendships among autistic children and young people has overlooked those with complex communication and learning needs, using methods that are not accessible to them. As a result, their views on friendship have often been underreported. We investigated the friendship experiences of 12 autistic adolescents, aged between 12 and 15 years, who have complex communication and learning needs and attend a specialist school. By using methods that were adapted including visual resources, the children were able to share their views on friendship. Our two main findings were (1) children identified friends who evoked strong emotions and connection, without distinguishing between positive and negative interactions or the standard boundaries of friendship; and (2) children’s descriptions of friendship communicated a preference for physical play in spaces that facilitated this. They also identified the need for space within structured settings. Our research gave the children the opportunity to identify their friends and express their preferences, offering important insights for future studies. These results highlight the value of supporting autistic children to build social connections by providing opportunities for active play and exploration of strong emotions within safe and familiar settings.

The diagnostic criteria for autism specify the presence of ‘deficits’ in social communication and interaction, including difficulties in making friends and/or the absence of interest in peers (American Psychiatric Association [APA], 2013). Furthermore, friendships have been identified as a particular area of challenge for autistic people since the earliest clinical descriptions of autism (e.g. Asperger, 1944; Kanner, 1943). Perceptions that autistic individuals do not seek social interaction have since progressed, with research suggesting that many autistic young people are socially motivated and desire friendships (Cresswell et al., 2019; O’Hagan & Hebron, 2016; Petrina et al., 2014; Sedgewick et al., 2016; Tierney et al., 2016). Friendships have been identified as an important way for autistic young people to develop socially, emotionally and cognitively (Cresswell et al., 2019; O’Hagan & Hebron, 2016; Sedgewick et al., 2018). Feeling accepted and having friends can also be important for wellbeing (Roffey, 2011), providing opportunities for autistic young people to socialise, share ideas, express emotions and experience emotional reciprocity (Cresswell et al., 2019). Crucially, while friendship is thought to aid in social development, this does not mean that autistic young people should be expected to socialise in the same manner as non-autistic young people. Rather, it is important to increase our understanding of the conditions under which friendship is experienced by autistic young people, including context-specific barriers and enablers of friendship.

Friendships have been defined as reciprocal relationships characterised by high levels of both knowing and liking (Newcomb & Bagwell, 1995). Newcomb and Bagwell (1995) described how, behaviourally, friendships involve positive engagement (e.g. talk, smiling, laughter), effective conflict management, shared task orientation and mutual affirmation and equality. However, studies examining autistic young people’s friendships report differences in the meaning and nature of these interactions. Many autistic young people describe their closest friendships as being less strong and less helpful than those of their non-autistic peers, while also reporting reduced intimacy in relationships and increased conflict (Bauminger & Kasari, 2000; Sedgewick et al., 2016, 2018). Despite many autistic young people desiring friendships, research has shown that they tend to report having fewer friends and reduced friendship quality compared to their non-autistic peers (Calder et al., 2013; Lasgaard et al., 2010; Locke et al., 2010; Mendelson et al., 2016; O’Connor et al., 2022; Sedgewick et al., 2019). Many autistic young people also tend to remain on the periphery of social networks, finding it difficult to initiate new friendships and understand normative social expectations, resulting in an increased risk of bullying (Cresswell et al., 2019; Fisher & Taylor, 2015). Such interactions can contribute to social withdrawal, isolation and feelings of loneliness (Grace et al., 2022; Sumiya et al., 2018).

Drawing on literature from the general population, friendships take on particular salience during adolescence, which is a time characterised by increased independence from parents and a stronger commitment to peer relationships (Blakemore, 2018). Several studies have identified that difficulties with making and maintaining friendships increase for autistic young people as they move through adolescence (Cresswell et al., 2019; Halsall et al., 2021; O’Hagan & Hebron, 2016; Tierney et al., 2016). These difficulties are attributed to various factors including school transitions, physical and emotional changes associated with puberty, increased awareness of autistic differences and increases in the complexity of social expectations.

Most research examining autistic adolescents’ friendships has focused on those who attend mainstream school provisions. In this context, school has been identified as a practical setting in which to support the development of friendships, with adults promoting structured opportunities for developing social interaction skills alongside non-autistic peers (Tierney et al., 2016). These opportunities may be particularly important, as autistic young people typically spend significantly less time involved in social interactions, both within and outside school, than their non-autistic peers (Bauminger et al., 2003, 2008). Calder et al. (2013) examined the friendship experiences of 12 pre-adolescent autistic children (aged 9–11 years) and found that while most reported satisfaction with their friendships, they described feeling lonely and excluded from their non-autistic peers, while also expressing a preference for playing alone at times. Within this study, parents and teachers were often aware of children’s preferences (i.e. that they found interacting uncomfortable and sometimes overwhelming), yet they felt compelled to encourage the children to socialise.

Such findings raise important questions about the implications of encouraging autistic children to conform to neurotypical social norms. While parents and teachers often regard socialising as desirable, children’s accounts highlight discomfort and a preference for solitude. This tension suggests that well-intentioned efforts to promote normative friendships may inadvertently undermine autistic children’s autonomy and identity, increasing the risk of stress and reduced wellbeing (Cage & Troxell-Whitman, 2019). Supporting friendships therefore requires balancing opportunities for connection with respect for autistic children’s preferences, ensuring these interactions feel authentic and manageable. Accordingly, the present study adopts an approach that seeks to document autistic adolescents’ own meanings of friendship and the environmental features that make such friendships viable.

Limited studies have examined autistic young people’s friendships within specialist schools. These provisions deliver education specifically for young people with special educational needs and disabilities, who are a variable cohort with high and complex communication and learning needs. There have, however, been two studies that used interview methods to examine the friendship experiences of autistic boys (Cook et al., 2016) and girls (Cook et al., 2018) attending either specialist or mainstream schools, together with their parents. The boys’ mothers described specialist schools as helpful for promoting friendships, due to small classes and supportive school staff, who actively facilitated activities to enable social interaction. They also described their sons’ friendships as improving following transition from mainstream to specialist school, with reduced bullying incidents. The girls from both school settings described their desire to have friends. However, parents noted that the girls’ social expectations, interests and ability to apply social skills were not keeping pace with their non-autistic peers. Across both studies, the autistic young people tended to befriend other autistic children or pupils with special educational needs, attributing this to shared interests and increased opportunities to feel accepted (see also Crompton et al., 2022). Both groups described experiencing isolation and bullying, which were more prevalent in mainstream schools. However, within specialist schools, the girls’ study highlighted the social challenges of their peers’ special educational needs, which contributed to friendship conflicts and experiences of bullying. These mixed findings illustrate a homophily paradox. Homophily is the tendency of individuals to associate with others who share similar traits (Banarjee et al., 2023; Messinger et al., 2019; Smirnov & Thurner, 2017). While shared neurotype, communication profile, or support needs can facilitate understanding and acceptance, heterogeneity within specialist cohorts may produce within-group boundaries that limit closeness.

Overall, these studies emphasise that friendship experiences among autistic young people in specialist school provisions are likely to be significantly different compared to those of their peers in mainstream education. It should be noted, however, that despite attending specialist school settings, the willingness and ability of these young people to engage in semi-structured interviews suggest a potential sampling bias. Within specialist school settings, there is a wide variation of presenting abilities and needs, including young people whose views can be difficult to access via traditional research methods. Research that is reliant on spoken communication (Cresswell et al., 2019; Fox et al., 2024; Petrina et al., 2014) is likely to preclude many autistic young people who attend specialist schools (Winstone et al., 2014), particularly those with complex communication and learning needs.

Among the few studies that have examined the friendship experiences of autistic children with co-occurring developmental disabilities (who may have complex communication and learning needs), findings were drawn from parental survey data. Studies by Taheri et al. (2021) and Solish et al. (2010) found that children with intellectual disabilities (IDs) and autistic children with ID participated less frequently in social activities, had fewer friends and experienced lower-quality friendships than their typically developing peers. Solish et al. (2010) also reported that autistic young people with ID participated in fewer social activities with peers and more with parents and other adults, limiting opportunities to develop reciprocal peer relationships.

Notably, these studies did not include the perspectives of young people themselves. This omission is problematic because parent-child reports often diverge on key domains including quality of life (Knüppel et al., 2018; Sandercock et al., 2020), autistic traits (Pagán et al., 2024), social skills (Cederlund et al., 2010; Lerner et al., 2012), executive functioning (Kenworthy et al., 2022) and symptoms of anxiety (Kaat & Lecavalier, 2015; Kalvin et al., 2020; Storch et al., 2012; White et al., 2012). Coupled with calls from autistic self-advocates to centre lived experience, there is a clear methodological gap: much of what is known about friendship among adolescents with complex learning and communication needs rests on adult-report measures that may not capture young people’s priorities (e.g. sensory comfort, shared routines or non-verbal reciprocity). Developing inclusive, multi-modal methods to elicit adolescents’ views directly, including approaches that empower non-speaking participants (Aidonopoulou-Read, 2025; Kenny et al., 2023), is therefore both a conceptual and ethical imperative.

Using inclusive methods of participation, we sought to address this gap in the literature. Specifically, we aimed to examine the friendships of autistic young people who attend specialist school provisions and have complex learning and communication needs. In addition to generating important data around friendships, the goal was to enrich the range of research approaches used to effectively elicit the views of this underrepresented group.

Our research questions were:

1. Can autistic adolescents who attend a specialist school and have complex communication and learning needs share their views around friendship, and if so, how?
2. What are the friendship experiences of autistic adolescents with complex communication and learning needs?
3. What facilitates or hinders friendship of these adolescents?

## Methods

### Participants

Purposive sampling was used to recruit participants from two campuses at Queensmill School, an autism-specific specialist school in London. Parents of autistic children were contacted via the school, and information was provided during parent coffee mornings. This sampling approach facilitated the inclusion of a breadth of communication and learning profiles within the school and increased the range of ethnicities, ages and genders within the sample. Fifteen parents were approached, with three children excluded due to not fitting the inclusion criteria (all below the desired age range). The final sample comprised 12 autistic children who met the inclusion criteria: (1) between 11 and 16 years old; (2) diagnosis of autism, with complex communication and learning needs and (3) attending specialist school.

The participants represented a range of ethnicities and included mainly boys, with one girl participating. This gender weighting is representative of the gender split within this age group in this specialist school. SCERTS (Social Communication, Emotional Regulation and Transactional Support; Prizant et al., 2014), which is used by the school, was used to provide context regarding each participant’s communication level. All participants were identified to be at the Language Partner (LP) level, which the school define as a child who uses symbolic means to communicate shared meanings with purpose (spoken word, sign language, picture symbols) and may use up to 100 spontaneous word combinations. Within this description, there is variability with the participants’ presenting communication, with some children identified as LP Minimal Spoken Language, communicating primarily via gesture, sound and picture symbols. Demographic details are reported in Table 1.

**Table 1.** Children’s demographics.

Pseudonym	Age (years)	Ethnicity	Gender	Communication level
Afan	12	Black African	Male	SCERTS LP Minimally verbal
Christano	12	Filipino	Male	SCERTS LP
Darius	12	Other Black Background	Male	SCERTS LP Minimally verbal
Ekele	13	Black Caribbean	Male	SCERTS LP
Eva	12	White British	Female	SCERTS LP Minimally verbal
Kabir	15	Indian	Male	SCERTS LP
Lyron	12	White Asian	Male	SCERTS LP Minimally verbal
Maceo	14	Black Caribbean	Male	SCERTS LP Minimally verbal
Omar	14	Black African	Male	SCERTS LP
Richard	12	White British	Male	SCERTS LP
Salman	13	White British	Male	SCERTS LP Minimally verbal
Thomas	14	White British	Male	SCERTS LP Minimally verbal

### Materials

Data-collection methods were developed by the research team. The team includes JH (an educational psychologist, former specialist teacher and parent of an autistic son with complex communication and learning needs, not associated with the participants’ school), LC and AC (academics specialising in autism research) and ER (a specialist drama teacher and middle leader within the participants’ school). The resources were piloted by AH (an autistic young person who attends a specialist college and has complex communication and learning needs). To support engagement and understanding for the participants, resources were developed according to key principles: including meaningful photos of people and places (e.g. photos of peers and school locations rather than generic symbols) alongside using visual resources already embedded within the school (to ensure familiarity). Prior to each interview, the resources were further reviewed using ER’s extensive knowledge of each child’s communication and learning profile, aiming to accommodate individual specific preferences to facilitate each child’s engagement (e.g. removal of red borders that were distressing for a specific child) (Appendix).

#### Photos of children and school staff

A collection of pre-existing photos depicting each member of the class (approximately eight children and six staff) were presented to each participant. These photos were developed for, and used by, the school as part of day-to-day classroom practice and were therefore familiar to the children. When key adults indicated that the participant showed interest in children from other classes, these were incorporated into the initial photos presented. The children also had the option to explore photos of children outside of their class, with these photos collated nearby.

#### Photos of spaces within school

Photos of familiar spaces within school (with names written underneath) were presented to the children. These photos showed outside spaces (playground and climbing equipment) and indoor spaces (classroom, music room, art room, lunch hall, soft play, sensory space, wet play and PE hall). The photos were personalised for each child depending on which school spaces were available to them.

#### Emotions

Resources showing visual images of faces depicting emotions and describing words were available to participants. The images were categorised using Zones of Regulation, a framework designed to develop self-regulation and emotional control (Kuypers, 2011). Each zone includes 8–14 emotions. Zones of Regulation is used within the school’s Personal, Social, Health and Economic provision, and the children are familiar with using these visuals to communicate their emotions.

#### Symbol of different types of play

Resources using Widgit (Widgit Software, 2024) symbols (simple images and key words) depicted different types of social interaction including playing, chasing, talking, laughing, holding hands, sitting with people, looking at people, high-fiving, having space or being close to people. Widgit symbols are used throughout the school and are a familiar approach to supporting communication for the children.

#### Friendship sentence starters

Resources including seven sentence starters in text and Widgit symbols exploring the children’s social interactions were available. Sentences included: ‘My friends are called . . . ’, ‘I like to play with . . . ’ and ‘When I see my friends, I feel . . .’.

#### Visual sorting activity

The visual sorting activity used 14 cards depicting social experiences. Each card showed a picture and written description (e.g. ‘sharing with my friend’, ‘sitting next to my friend’ and ‘when my friend doesn’t want to play’). Cards were sorted using thumbs up/thumbs down or by placing them on Zones of Regulation emotions.

### Procedure

Interviews were completed in a quiet, familiar and private room within the children’s school. Each interview was led by ER (who was familiar to the children), supported by JH (who was working with these children for the first time). Interviews began with consent and rapport-building, particularly to introduce them to JH. Interviews lasted between 6 and 17 minutes, after which JH and ER had a short debrief, a process that supported contextual understanding and facilitated reflexivity and data interpretation. All children followed the same initial procedure of settling into the room, with time provided to build rapport and confirm consent. The duration and support required to build rapport varied for each child and included the adults engaging with transitional objects the child had brought to the room with them, which often centred on their sensory needs and interests. Rapport-building occurred during the initial part of the interview and took between 2 and 6 minutes.

**Figure 1.**
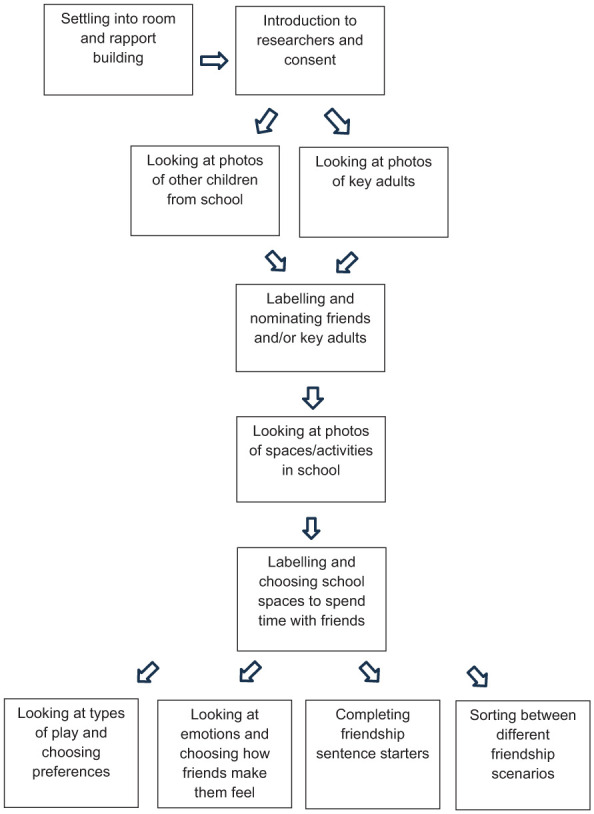
Interview procedure.

Following this rapport-building phase, each child accessed a selection of interview resources, adapted to their communication, social preferences and presentation within the interview context. Children were presented with photos of other children and/or photos of key adults, which they were encouraged to label and choose their preferences from (e.g. ‘who can you see?’, ‘who is your friend?’ and ‘I like to play with . . .’). All the children then examined, labelled and chose preferences from photos of activities within the school (e.g. ‘what do you like to do?’, ‘what do you like to do with your friend XX?’ and ‘when XX and I go to the playground I feel . . .’). Seven of the children continued with the interview and accessed a selection of the remaining resources, chosen according to their communication needs and presentation within the room. Details are presented in Figure 1.

### Ethics

Research involving autistic adolescents with complex communication and learning needs presents distinctive ethical challenges, which we summarise in detail here.

Many young people in our sample relied on alternative or augmentative communication systems, had learning profiles that could affect their understanding of research information and encountered sensory or environmental barriers that could impact on their ability to engage. These factors, coupled with potential inequalities in decision-making power, meant that careful planning and reflection were essential for ensuring that participation was meaningful, comfortable and respectful.

The study was conducted in line with the Declaration of Helsinki, and ethical approval was obtained from the Research Ethics Committee at UCL Institute of Education, University College London. All standard ethical expectations for psychological research (e.g. right to withdraw, anonymity) were followed (British Psychological Society, 2014). Consent was initially obtained from the specialist school, who subsequently facilitated contact to gain informed written consent from each parent, before children were approached. Parents were encouraged to discuss the project with their children. Children’s consent was sought during the research process, as this was judged by the school and parents to be most meaningful to them.

Careful consideration was given to supporting the children’s understanding of informed consent. We designed simple visual consent forms, shared at the beginning of the study, and reviewed with the children. The children’s assent to participate was carefully monitored throughout the process by both of the researchers, who carefully looked for changes in body language, communication and engagement. The researchers used these observations, alongside frequent check-ins using familiar school approaches, such as choices (e.g. ‘more?’ or ‘finished?’), to monitor ongoing consent.

Consistent with participatory and neurodiversity-affirming approaches (Pellicano & den Houting, 2020), development of the interview resources was collaborative, drawing on the expertise of the research team, including an autistic advisor with similar communication needs, who supported the development of the materials to ensure they were meaningful and respectful. To gather contextual information regarding the children’s social interactions, brief informal observations of the children during a social activity (e.g. playtime) were completed by ER. These observations were shared with JH prior to the interviews and used to inform which resources were likely to be meaningful to the children.

The photos used within the resources were sourced from pre-existing visuals, which showed a photo of each member of the class. These photos are used widely throughout the school to support children’s understanding and recognition of peers. Parental consent for using these photos within the research had previously been secured via the school’s wider consent for obtaining and using photographs.

Prior to each interview, JH and ER discussed the children’s communication preferences and adapted interview resources to support them to participate. Throughout each interview, the researchers monitored participants’ comfort, engagement and consent, adapting and/or finishing the activities accordingly. Debrief discussions after each session supported reflexivity and helped identify any emerging ethical concerns.

### Analysis

Interviews were audio recorded and transcribed, and photographs of the resources were taken for each child. Detailed notes were taken, describing each child’s body language and non-verbal interactions, including pointing, gesture, eye contact and changes to shared interaction. These notes were analysed concurrently with audio responses. The analysis adopted an inductive approach, identifying patterns within the data without intending to integrate these within pre-existing concepts and preconceptions regarding friendships. The analysis was informed by a social constructionist perspective, which recognised that experiences of friendship will be conceptualised and understood differently according to each participant’s communication and learning profile, as well as their individual experiences. Data were analysed using reflexive thematic analysis (Braun & Clarke, 2021). This process included recursively cycling through the stages of (1) data familiarization; (2) code generation; (3) developing themes; (4) review and development of themes; (5) naming, refining and defining themes and (6) write-up.

During code generation, codes were generated from both transcripts of audio responses and descriptions of the participants’ non-verbal interactions such as ‘repeatedly pointed to the photo to indicate preference’. During subsequent stages, care was taken to ensure equal priority was shown to all types of communication, ensuring the analysis accurately depicted the experiences of all children, and that verbal and non-verbal responses received equal consideration.

The research team’s understanding of autism is influenced by a neurodiversity lens, through which autism is interpreted as a natural and valuable aspect of human variation, as opposed to a problem or deficit in need of ‘fixing’ or ‘changing’ (Pellicano & den Houting, 2022). A hermeneutical approach was used to provide transparency and clarity regarding the researchers’ assumptions when interpreting the participants’ narratives (Gadamer, 2004). The analyses were led by JH, in consultation with the other authors, who reviewed and refined themes.

Reflexivity was actively engaged throughout the data collection and analysis, utilising one researcher’s (JH) experience in supporting and interpreting the views of autistic children with complex communication and learning needs, and another researcher’s (ER) extensive experience working with the specific children involved in this study. Debriefing between JH and ER following each interview provided context to the children’s communication, which informed interpretation. These interpretations were revisited regularly in whole team discussions, where assumptions were questioned and the meaning of the children’s spoken, visual and body language was considered. This process included shared reflection on how the themes incorporated the range of communicative responses. This reflexive process also aimed to recognise and provide transparency regarding the influence of neurotypical assumptions on the interpretation of the data. Throughout the process, a note of discrepancies and inconsistencies was kept and used to identify any perspectives that did not align within the neurodiversity lens of the research team, leading to further development and refining, to enhance rigour.

## Results

**Figure 2.**
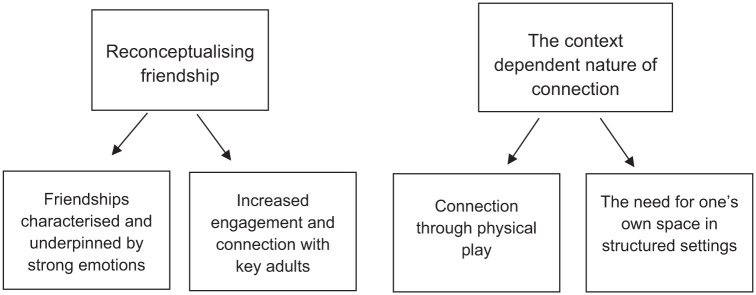
Thematic map.

Themes and sub-themes are depicted in Figure 2. All names have been replaced with pseudoyms.

### Theme 1: reconceptualising friendship

All the children showed familiarity with, or were able to label, their classmates. The majority of children nominated at least one friend and were often able to share their views regarding friendship. The children’s identification of friends appeared to be driven by (1) friendships characterised and underpinned by strong emotions and (2) increased engagement and connection with key adults.

#### Sub-theme 1: friendships characterised and underpinned by strong emotions

All children identified friendships that were characterised by strong emotions. Some children expressed positive emotions about how their friends made them feel, using words such as good, okay and happy. For example, Chrisanto discussed his connection with a classmate, Sarina:

Researcher: When you are with Sarina, how does Sarina make you feel?

[Chrisanto looking at emotions . . . looking at each colour before pointing to good]

Chrisanto: Good

Researcher: Good

Chrisanto: Calm, excited [pointing to the green emotion zone]

We observed that several children communicated their emotional responses through gesture and movement, sounds and facial expression. Our interpretations treated these cues as equally meaningful indicators of connection, which were understood using the researchers’ understanding of the children’s individual communication approaches. For example, Ekele moved his face close to specific pictures, pointing and holding these, turning to share attention with the researcher and pushing the picture to their face.

Yet none of the children differentiated between friends who made them feel good and friends who evoked more challenging emotions. Indeed, some of the children expressed less positive, but equally strong, emotions towards the children they had identified as friends. Through reflexive discussion, we recognised that these expressions were not necessarily contradictions but reflected how the children experienced friendship as emotionally intense, rather than exclusively positive. This interpretation was supported by consistent patterns across participants, where high emotional arousal appeared central to their sense of connection and social interest. For Omar, this included an example of when feeling excited progressed into feeling out of control:

Researcher: How does Lucia make you feel?

Omar: Excited. Out of control [also pointing at images that depicted these emotions]

Likewise, one child noted that a chosen friend made him feel scared, while another child described feeling depressed about one classmate and jealous of another:

Researcher: What about David, how does David make you feel?

Richard: Depressed

Researcher: . . . alright and how does Theo make you feel?

Richard: Jealous

Several children identified friends as people that they have limited contact with but still felt strong emotions towards. These strong emotions included excitement about children they only saw for limited periods, often within shared play spaces in school:

Researcher: Choosing [researcher prompting Afan to choose from the pictures]

Researcher: Who are your friends?

[Afan taking the researcher’s hand and pointing to each child as the researcher names them, with repeated pointing to self. Change in Afan’s body language as he points to Elijah and Oliver, who are older pupils from a different class]

Researcher: Yes that’s Elijah and Oliver

[Lots of pointing to Oliver with increased eye contact to researcher. Afan laughing and pointing, increase in shared attention]

Researcher: Oh yes, that’s Oliver and you like to play with Oliver don’t you?

[Afan appearing excited and laughing]

Sometimes, the identification of other children appeared to be motivated by curiosity because the person was not in the place that the children expected, or had previously been part of their class and had now moved on:

Researcher: Salman, who is your friend?

[Salman looking at photos of children from their class. Salman pulling out different photos that were to the side and featured children from other classes]

Salman: Charmaine [pointing to child that moved to a different class]

Researcher: Charmaine, yes she is missed.

Salman: Charmaine

Researcher: Yes, Charmaine used to be in your class.

Salman: Yes

Researcher: Ok and looking at all the pictures Salman, who is your friend?

Salman: [pointing again to Charmaine, and Rowan]

Some of the children’s identification of friends appeared to be motivated by the excitement of conflict and challenging interactions within the class, which often resulted in children being kept separate:

Kabir: Faizan

Researcher: Faizan, yes, where? Show me where’s Faizan?

Kabir: There [pointing to Faizan]

Kabir: Bop [poking Faizan’s nose in picture]

Researcher: Yes, you do sometimes bop. Do you bop Faizan?

Kabir: No, don’t bop Faizan.

Researcher: And if you had to choose a friend, who would you choose?

Kabir: Faizan.

Researcher: Faizan, ok and what would you do with Faizan?

Kabir: [pointing to playground visual]

Researcher: You would play in the playground?

Kabir: Playground

For one of the older children, the strong emotions associated with adolescence appeared to have become intertwined with friendship. Their awakening interest had to be boundaried and guided by the school staff.

Researcher: I like to play with . . .? [researcher paused] Who do you like to play with?

Omar: Lucia

Researcher: Lucia. Ok, you like to play with Lucia

Omar: No touch Lucia

Researcher: No touch Lucia

Omar: Lucia touch girl

Researcher: Yeah

Omar: Lucia, no more touch

Researcher: No touch Lucia

Omar: No touch girls

In reflecting on this interaction, we were mindful that these exchanges could be interpreted simply as behavioural guidance around physical boundaries. However, through reflexive team discussions, we considered Omar’s responses as demonstrating a developing awareness of social rules and friendship expectations linked to adolescence. This interpretation aligns with the broader theme of friendships underpinned by strong emotions, highlighting how emerging self-awareness and social curiosity were expressed and negotiated within the safety of the school context.

#### Sub-theme 2: increased engagement and connection with key adults

Most of the children named one or more adults they had a close connection with. A few children, who presented with minimal communication (primarily via gesture, sound and picture symbols), showed less interest in peer friendships, instead demonstrating increased engagement and connection with key adults.

Researcher: [puts out pictures of children Maceo spends time with]

Researcher: Who can you see?

Maceo: [Briefly looking at pictures and lifting these so they fall]

Researcher: Maceo, who are your friends?

Maceo: [Pointing to each picture in turn as researcher labels them]

Researcher: Maceo, who are your friends?

Maceo: [Pointing to each picture and then move away from table, playing with playdoh in a bowl for couple of minutes]

Researcher: [Puts out pictures of key adults alongside children. The adult pictures capture Maceo’s attention, and he quickly moves close to the table to look at these intently]

Researcher: Shall we choose one?

Maceo: [Immediately pointing at Sami (key adult). Change in body language, appearing animated and smiling, holding picture of Sami up]

Researcher: Sami

Maceo: [Pointing repeatedly at Sami before moving away from table and jumping up and down, smiling. Pause for nearly 2 minutes before choosing to move back to the table]

Researcher: [All pictures remaining on table] Ok Maceo, who is your friend?

Maceo: [Immediately picked up photo of Sami and handed it to the researcher]

In interpreting these moments, we reflected on how the participants’ responses were shaped by the adults’ dual roles as both caregivers and communication partners. Our familiarity with the school context helped us recognise that these adult–child relationships often provided a scaffold for emotional safety and social reciprocity, forming a bridge towards wider peer interaction. When asked to choose a friend, one child selected only his key adults:

Researcher: I want to ask again, who are your friends?

[Ekele leaning across to pull out the picture of staff which was underneath the photos of classmates and looking closely at Pat]

Ekele: Pat

Researcher: Is Pat your friend?

Ekele: Pat

[Ekele smiling and showing increased engagement]

Through reflexive discussion, we considered whether these expressions should be viewed as signs of social dependence or genuine markers of friendship. We concluded that, for several children, key adults represented trusted relational partners through whom comfort, regulation and social learning occurred. This interpretation reflects our reflexive stance that friendship, for these children, was communicated through emotional attunement, comfort and shared presence, rather than measured against neurotypical ideals of reciprocity.

### Theme 2: the context-dependent nature of connection

Within the children’s descriptions of friendship, it was evident that the type of connection they preferred was dependent on the context. Within this theme, two sub-themes were identified: (1) connection through physical play and (2) the need for one’s own space in structured settings.

#### Sub-theme 1: connection through physical play

When asked where in the school they would like to go with their friends, all the children indicated a preference for active spaces, which facilitated physical play and had equipment to climb on:

Researcher: So Eva, where do you like to go with Richard?

Eva: Playground, playground

The majority chose outdoor spaces, and none of them chose structured learning environments, such as the classroom.

Researcher: You like to spin [your friend] Oliver

[Afan laughing, with eye contact and some copying of spin actions]

[Afan turning in chair and lifting t-shirt (interpreted by researcher as tickling gesture)]

Researcher: Yes and you like to tickle him

Afan: bobobobaba [unclear sounds, louder and sounding excited and happy, lots of smiles and increased shared attention]

Several children described their preferred type of play, which involved physical interaction such as chasing, tickling and rough and tumble with friends (including both peers and adults).

Researcher: So Thomas, when you are with Jacob and Nathan, what do you like doing?

[Thomas pointing immediately to playing, chasing]

Researcher: You like playing, chasing

Across participants, these physical exchanges appeared to facilitate shared connection and enjoyment. Our reflexive discussions highlighted how active play, often overlooked in verbal definitions of friendship, represented meaningful social participation for children whose communication was primarily through non-verbal means. We remained mindful that interpreting these moments through a neuro-normative lens could obscure their relational significance. Instead, we recognised that for these children, friendship was often experienced through movement and sensory synchrony rather than through conversation or mutual disclosure.

#### Sub-theme 2: the need for one’s own space in structured settings

Some children highlighted that they preferred to have space from their friends and did not want to sit next to them or be in a group together.

Researcher: So do you like being on your own?

Salman: Yes

Researcher: Do you like sitting next to Rowan?

[Salman pointed clearly to thumbs down and shaking his head very strongly]

Researcher: No

This preference for space appeared to be focused around times that the children were in structured learning spaces, such as the classroom:

Researcher: So when you are with Dayson, where do you like to go?

Richard: Playground [said with urgency]

Researcher: Uh huh, yeah. Is there anywhere else you like to be with your friends?

Richard: [Looking at pictures of places around school]. Playground, soft play, playground. [Richard picked up playground symbol and passed to researcher]

Researcher: So when you are in the playground, what do you like doing?

Richard: Like chasing [also pointing to the chase symbol]

Researcher: Chasing . . . ok and who do you like chasing in the playground?

Richard: Chasing Dayson [also pointing to Dayson]

Researcher: You like chasing Dayson, and when you are inside [researcher pointing to classroom visual], what do you like to do with Dayson?

Richard: Space [also pointing to space symbol]

Researcher: You like playing with Dayson outside and space from Dayson inside?

Richard: Space inside

Through reflexive discussion, we recognised that the children’s references to space reflected their desires to regulate sensory and emotional demands, particularly within structured settings. As practitioners who were (or who became) familiar with the school environment, we were mindful to interpret these preferences as active expressions of comfort and autonomy rather than absence of social interest.

## Discussion

There is growing awareness of the important role that friendship has in the lives of autistic children and young people, with many valuing shared interests and companionship (Fox et al., 2024). However, minimal research has examined the friendship experiences of autistic children and young people in specialist schools, especially those with complex communication and learning needs. Where research was previously undertaken with this group, their views and experiences were primarily collected via indirect methodologies, such as researcher observation and/or parent/educator report (e.g. Bradley & Male, 2017; Fox et al., 2024). Therefore, our research sought to gather the views of autistic adolescents with complex communication and learning needs who attend specialist school, eliciting crucially important firsthand experiences and perspectives regarding their friendships.

There are many challenges inherent with gathering the views of this underrepresented group, particularly regarding an abstract concept such as friendship. Given that reflexive thematic analysis is grounded in a hermeneutic approach, we were mindful of the potential for bias if responses were interpreted through a neurotypical lens, as preconceptions about friendship could shape the analysis. To mitigate this risk, it was considered fundamental to focus exclusively on the children’s views, rather than collecting those of the key adults that support them. This decision was informed by literature that highlights how the views of key adults often do not align with autistic individual’s experiences of friendship (e.g. Calder et al., 2013; Fox et al., 2024), alongside a recognition of power dynamics within adult/child and disability narratives. Trusting the authenticity of the children’s responses was therefore central to our approach.

The children within the study communicated different levels of engagement and understanding regarding their concept of friendship, and for some children, it was not possible to fully ascertain their understanding of this concept. However, supported by inclusive and flexible methodologies, all the children communicated their preferences regarding the interactions and contexts that promoted connection. This information provides an important starting point for understanding their experiences of friendship.

Our first theme indicated that the children’s friendships were characterised by interactions and connections that evoked strong emotions for them. Within their nominated friendship, the children appeared to be drawn to the strength of the emotion experienced, rather than being motivated solely by the positive emotions that are generally connected to friendships. This understanding of friendship may reflect the specific context of this specialist school, with positive intent often ascribed to children’s actions even when behaviours are perceived as challenging, and adults referring to all children within a class group as ‘friends’ without differentiating according to individual relationships. These patterns echo findings from Cook et al. (2016, 2018) and Halsall et al. (2021), who also noted that specialist environments can foster safety and belonging while shaping how friendship is expressed and supported. The limited research that has examined friendship experiences in specialist and mainstream schools has described the complex impact that other children’s communication and regulation needs can have on building friendships, noting that challenging interactions may be considered less intentional within specialist schools than mainstream settings (Cook et al., 2018; Fox et al., 2024). This experience may confuse the boundaries of friendship. The different social experiences and associated social expectations in specialist schools are also likely to underpin the children’s close connection to key adults, which was sometimes interpreted as friendship by the children. Within the specialist school context, key adults have been recognised as having multiple roles, including meeting the children’s basic needs (Rees, 2024), which can result in greater dependency and provide an initial bridge to promote early social interaction.

Our second theme highlighted the importance of active play and shared activities for facilitating connections. Previous research has described autistic children’s preferences for companionship, with a focus on activities centred around shared interests (Calder et al., 2013; Finke et al., 2023; Fox et al., 2024). Pritchard-Rowe et al. (2024) examined autistic adults’ play experiences and emphasised the value of play as central to wellbeing, identity and emotional regulation. They described the recuperative experience of solitary play, while also recognising the value of social play for building connection (especially social play with other autistic individuals). These findings highlight the diversity and individuality of autistic play experiences, challenging neurotypical assumptions that prioritise highly social forms of play. Pritchard-Rowe et al. (2024) also highlight the value of social connections alongside the potential exhaustion they can bring, reinforcing the need for balance and recovery. Although the children in our study could not verbalise such experiences, their shifts between energetic physical play and the preference for space suggest a similar rhythm of connection and recuperation. Drawing on this broader literature allows us to recognise that the wish for social connection may naturally coexist with a need for sensory and emotional regulation, both of which are authentic and adaptive aspects of friendship for this group.

Other research examining contexts that facilitate friendships for children with complex needs has highlighted the role that active and fun play, as well as and drama opportunities, can provide for building friendships and peer connections (Bradley & Male, 2017; Robinson & Crane, 2025). Within the current study, similar findings were apparent in the children’s preferences for participating in physical activities with their friends, supported by their key adults. The children showed a preference for active play that incorporated sensory input with social interaction, and they communicated positive feelings associated with this play. It is notable that when asked about places in which they would choose to spend time with friends, none of the children chose a structured learning context. Indeed, within the classroom context, several children expressed a preference for space and desired not to sit next to friends. This social preference may be informed by the children’s sensory needs, which are recognised as a core aspect of autism, and the need for additional space may provide a method for managing the demands of complex needs within a structured environment. This desire for space is reflected in previous research exploring autistic young people’s views regarding their friendships, with children feeling overwhelmed by expectations of needing to constantly socialise and preferring to spend some time alone (Calder et al., 2013).

Alongside the valuable contribution this research provides in examining friendship for an underrepresented group in the literature (Russell et al., 2019), this work also presents a crucially important contribution to developing inclusive and accessible methodologies. Directly accessing children’s views is important, particularly when examining a concept such as friendship, where adults’ views have been identified as differing from their children’s understanding of friendship (Calder et al., 2013; Fox et al., 2024). Inclusive research methods have been identified as a key area of focus (Cascio et al., 2021; Lewis-Dagnell et al., 2023; Parsons et al., 2021), with autistic children who have complex communication and learning needs often being excluded from research due to priority being given to spoken interviews and/or information gathered using indirect approaches. The current study adds to a limited, but emerging, body of literature (Bradley & Male, 2017; Doak, 2019; Lewis-Dagnell et al., 2023; Parsons et al., 2021) providing insight into the approaches that support children with complex communication and learning needs to share their experiences. An important facilitator was close collaboration with key adults who were already familiar to the children, enabling them to feel regulated to participate, their communication to be interpreted, and interview resources to be adapted according to their presenting needs and preferences.

Our findings provide several critically important implications for supporting children’s friendships within special schools. A key implication is that by using visual resources already embedded in the school, the children were able to express their social preferences regarding friendships, including activities and sensory experiences they preferred and their associated emotions. This insight into the children’s preferences provides important information regarding contexts that their key adults can use to promote the children’s social engagement and development of connections with their chosen peers.

A second implication regards the importance of facilitating opportunities for children to build connections through active, physical play. Such approaches can help to support the children’s sensory needs, while also providing connections centred around fun and shared activities, which can be used to promote positive interactions that can develop into friendships.

A final implication is that strong emotions (whether positive or negative) appear to underpin the children’s interest in social connections. This finding suggests that friendships for children in specialist settings may be different from a neurotypical concept of friendship. The double empathy problem reflects the mutual misunderstandings that commonly arise between individuals of different neurotypes (e.g. autistic vs non-autistic), due to a lack of shared understanding (Milton, 2012). As such, differences in social interactions and preferences should be understood in parallel with the challenges non-autistic individuals have in understanding and adapting to the social interaction and communication styles of autistic people. This perspective is particularly important to be aware of when the children have such a strong dependence upon their key adults. It is therefore important that adults carefully consider the language they use to describe or define friendships to the children they are supporting.

Finally, it is important to note the limitations of the current research. First, there were a number of factors that needed to be balanced to facilitate each child’s ability to engage in the research and to support their understanding of the abstract concept of friendship. One limitation with only gathering the children’s direct views meant that it was difficult to establish their understanding of the concept of friendship or whether they were nominating peers according to different priorities. Although this limitation impacts the extent to which their responses can be characterised within a neuro-normative interpretation of friendship, their communications about their experiences and preferences provide helpful indications regarding the children’s feelings of connection and contexts that facilitate this with the people that they nominated.

A second limitation regarded the necessity to use visual resources that were embedded within the school context and familiar to the children, limiting these sufficiently so the children did not become overwhelmed. While this approach supported engagement and comprehension, it may have limited the children’s ability to express views that were not included within the predetermined visuals, such as friendships outside of their school context. These limitations were addressed, to an extent, by a member of the research team (ER) being familiar with the children’s communication style and social preferences within school and prioritising a selection of visual prompts that were personalised for each child, while also including a broader range of visuals (including all children and key adults in school) and encouraging children to explore these if they showed engagement with them.

## Conclusion

This study examined the friendship experiences of children attending specialist schools, gathering the direct views of autistic adolescents with complex communication and learning needs. The study provided critically important insights into the contexts that facilitate connection for these children, providing an important starting point for understanding their experiences of friendship. Many of the children showed an interest in interactions and experiences that evoked strong emotions, without distinguishing between situations that were more or less positive or adhering to the standard friendship boundaries and social expectations. Connection with peers was also facilitated by opportunities for active play focused around the children’s interests, while a need for space and quieter time was identified.

These insights provide important guidance for specialist school staff to expand the children’s friendship interactions, ensuring that opportunities are provided to develop connections outside of structured interventions based on neuro-normative interactions and greetings. These should include facilitating children to participate in active and motivating activities alongside peers, which incorporate their interests and evoke strong emotions within a safe and contained context.

In addition to these findings, this study contributes to an emerging body of research on inclusive methodologies that can effectively engage children with complex communication and learning needs. By using familiar visual resources, personalised prompts and a familiar trusted adult, we were able to bridge communication barriers and gain insights into the children’s preferences and experiences. This approach offers a valuable model for future research and practice, ensuring that the voices of children with complex communication and learning needs are centred in the development of interventions and educational practices designed to meet their needs.

## Appendix of resources

Due to confidentiality, the first two resources are described but are not visually included. The remaining resources were drawn from the school’s social and emotional curriculum resources. Due to copyright restrictions, these are described but not visually included. They are used throughout the school to support children to develop understanding, express preferences, interests and emotions and are therefore familiar to the children.

### Photos of children and school staff

A collection of pre-existing photos depicting each member of the class (approximately eight children and six staff) were presented to each participant. These photos were frequently used within the classroom and were familiar to the children. When key adults indicated that the participant showed interest in children from other classes, these were incorporated into the initial photos presented. The children also had the option to explore photos of children outside of their class, with these photos collated nearby.

### Photos of spaces within school

Photos of familiar spaces within school (names written underneath) were presented to the children. These photos showed outside spaces (playground and climbing equipment) and indoor spaces (classroom, music room, art room, lunch hall, soft play, sensory space, wet play and PE hall). The photos were personalised for each child depending on which school spaces were available to them.

### Emotions

Resources showing visual images of faces depicting emotions and describing words were presented to the children. The images were categorised using Zones of Regulation, a framework designed to develop self-regulation and emotional control (Kuypers, 2011). Each zone includes 8–14 emotions. Zones of Regulation is used within the school’s Personal, Social, Health and Economic provision, and the children are familiar with using these visuals to communicate their emotions.

### Symbol of different types of play

Resources using Widgit (Widgit Software, 2024) symbols (simple images and key words) depicted different types of social interaction, including playing, chasing, talking, laughing, holding hands, sitting with people, looking at people, high-fiving, having space or being close to people. Widgit symbols are used throughout the school and are a familiar approach to supporting communication for the children.

### Friendship sentence starters

Resources including seven sentence starters in text and Widgit symbols exploring the children’s social interactions. Sentences included: ‘My friends are called . . .’, ‘I like to play with . . .’ and ‘When I see my friends, I feel . . .’.

### Visual sorting activity

The visual sorting activity used 14 cards depicting social experiences. Each card showed a picture and written description (e.g. ‘sharing with my friend’, ‘sitting next to my friend’ and ‘when my friend doesn’t want to play’). Cards were sorted using thumbs up/thumbs down or by placing them on Zones of Regulation emotions.
